# fMRI Investigation on Gradual Change of Awareness States in Implicit Sequence Learning

**DOI:** 10.1038/s41598-017-16340-2

**Published:** 2017-12-01

**Authors:** Jianping Huang, Yingli Li, Jianxin Zhang, Xiangpeng Wang, Chunlu Huang, Antao Chen, Dianzhi Liu

**Affiliations:** 1 Department of Psychology, Soochow University, Suzhou, 215000 China; 2 Department of Psychology, Tsinghua University, Beijing, 100084 China; 3 Key Laboratory of Cognition and Personality of Ministry of Education, Faculty of Psychology, Southwest University, Chongqing, 400715 China

## Abstract

Awareness of implicit knowledge is a changing process. Previous studies have examined brain activation patterns corresponding to the start and end stages of implicit learning, but failed to reveal the gradual changing course of awareness in implicit learning. The present study explored brain activation changes corresponding to different awareness states elicited by two different stimulus onset asynchrony (SOA, 850 ms and 1350 ms) over the whole course of implicit sequence learning (i.e., divided into three phases), by using a process dissociation procedure (PDP) paradigm and the technique of functional magnetic resonance imaging (fMRI). In the results, it was found that the 850 ms SOA elicited primarily an awareness state of unconsciousness, under which the frontal lobe was significantly activated during the early phase of implicit sequence learning, with its activation levels correlated positively to consciousness levels. In contrast, the 1350 ms SOA triggered predominantly an awareness state of consciousness, under which the activation levels of the inferior parietal lobule correlated positively to consciousness levels during the middle phase, and positively to consciousness levels as well as negatively to unconsciousness levels during the late phase of implicit sequence learning. Overall, the frontal lobe and inferior parietal lobule were found to play critical roles in mediating awareness states over the course of implicit sequence learning.

## Introduction

Implicit learning is involved in many fundamental skills, such as language acquisition, motor training, and social behavioral learning^1,2^. The function of conscious and unconscious processing in implicit learning has been an intriguing topic for many psychological researchers^3–5^. In the literature, controversy exists regarding whether consciousness and unconsciousness are dissociated or collaborated during a learning process. Some researchers hold an all-or-none viewpoint on this issue, and argue that knowledge obtained from any learning process is either conscious or unconscious. According to this line of thinking, implicit learning is regarded as a completely unconscious processing, as opposed to explicit learning, which involves only conscious processing^6–9^.

However, other researchers insist on a dynamic gradual change viewpoint that consciousness and unconsciousness constitute a continuum, and participants’ awareness gradually changes on the continuum from unconsciousness to consciousness in the course of implicit learning^10–13^. Consistent with this viewpoint, Destrebecqz and Cleeremans used the process dissociation procedure (PDP) to measure awareness states in different response-stimulus-interval (RSI) settings during implicit learning, and found that awareness of the implicit knowledge was improved from complete unconsciousness in a 0 ms RSI condition to partial consciousness in a 250 ms RSI condition^14,15^. Based on these results, they proposed a representation quality theory that representation quality of implicit knowledge improved with elongated RSI, and that the better the representation quality, the more conscious the implicit learning process became^16^. In a review study, Norman found that the use of different measures or designs resulted in different magnitudes of implicit learning, and concluded that this result could not be explained by an all-or-none viewpoint on the relationship between consciousness and unconsciousness in implicit learning, but could fit into the dynamic gradual change viewpoint^17^.

Similarly, fMRI literature suggests that a dynamic gradual change viewpoint may be more proper than an all-or-none viewpoint for explaining the relationship between consciousness and unconsciousness in a learning process. Despite that sometimes neural dissociation between the two mechanisms can be observed in some perceptual-motor skill studies^18^, it has been reported in many fMRI studies that conscious and unconscious processes activate the same brain regions such as medial temporal lobe^19,20^, or share the same functional brain circuit (i.e., prefrontal cortex, striatum, anterior cingulate cortex, and visual cortex)^21^. Moreover, some researchers have found brain activation patterns corresponding to gradually improved representation in the process of implicit sequence learning^22–24^. For example, Gheysen and colleagues investigated brain activation patterns in early and late stages of implicit sequence learning by using fMRI and found that activation in left anterior hippocampus significantly correlated with the behavioral learning progress^23^. Ashe and colleagues found in their fMRI study that motor cortex mediated the early stage processing and frontal lobe mediated the late stage processing of implicit learning, suggesting that the early and late stages of implicit learning were supported by two types of processing differed in awareness states, with the former involving more unconscious processing and the latter more conscious processing^22^. It should be mentioned that most of these pertinent studies focused on brain activation changes corresponding to early and late stages rather than the whole process of implicit learning, which made it impossible to provide brain functioning evidence of gradual changes of awareness in implicit learning. For research on the gradual development of awareness in implicit learning, an fMRI study comparing brain activation patterns corresponding to different levels of awareness (i.e. defined by valid awareness measures) in implicit learning may be more useful.

The process dissociation procedure (PDP) is a well-established mechanism for differentiating and quantifying the relative contribution ratios of consciousness and unconsciousness in a learning process^25^. In a previous study, Destrebecqz and colleagues used the PDP to successfully separate conscious and unconscious components in both implicit and explicit learning processes^26^. In a similar way, the PDP was used in the present study as a tool to measure conscious and unconscious components in the process of implicit sequence learning.

To date, no previous study has investigated brain activation patterns corresponding to gradual changes of awareness in implicit sequence learning and thus brain regions mediating different stages of implicit learning process have never been fully understood, which were explored in the present study by using the technique of fMRI. In the present study, the PDP was adopted to quantify awareness states, and then correlation analyses between brain regional activation levels and corresponding awareness scores for each training phase (i.e., early, middle and late phases) of the implicit sequence learning process were conducted. It was hypothesized that certain core brain regions suggested by previous studies such as putamen^27–29^, frontal lobe^30,31^, and inferior parietal lobule^32^ would continuously mediate the whole implicit learning process, with their activation levels varying in accordance to awareness changes measured by the PDP.

## Methods

### Participants

Fifty university students participated in the present study. All participants were right-handed and mentally healthy, with normal or corrected-to-normal vision. None of the participants had previous experience with implicit learning experiments. All subjects gave written informed consent in our experiment. They were informed of their right to withdraw at any time. They received a small amount of payment after finishing the test. The study was approved by the Human Research Ethics Committee of Soochow University (China) and was in accordance with the ethical guidelines of the Declaration of Helsinki.

Four participants’ data were not used in the final data analysis because of low behavioral accuracy (<90%, following the criterion in Weiermann’s study^33^) and one participant’s data were excluded because of significant head movement. Twenty-three of the remaining participants (8 males and 15 females) were allocated in the 850 ms SOA condition, and twenty-two (7 males and 15 females) were in the 1350 ms SOA condition.

Prior analysis on participants’ background information showed that the two groups of participants did not differ significantly on general IQ levels (measured by the Raven’s Advanced Progressive Matrices) or other demographic variables (e.g., age and SES status) (see Table 1).

**Table 1:** Comparison of IQ levels and demographic variables between the 850 ms and 1350 ms SOA group.

	850 ms SOA group M (SD)	1350 ms SOA group M (SD)	*t* tests
IQ (accuracy)	0.62 (0.07)	0.64 (0.09)	*t*(43) = −0.86, *p* > 0.05
Age	21.30 (1.92)	20.82 (1.61)	*t*(43) = 0.89, *p* > 0.05
SES (1–7 scale)	3.48 (0.89)	3.36 (0.95)	*t* (43) = 0.42, *p* > 0.05

### Materials

Four blue circles (diameter = 4.6 cm, distance between the centers of every two adjacent circles = 6.9 cm) were presented horizontally on a computer screen. The target was a solid circle and the other three were hollow circles. Participants were required to press a key corresponding to the target as quickly and as accurately as possible. The implicit sequence order for the spatial locations of the targets was a second-order conditional (SOC) rule: 342312143241. In a SOC rule, the location of each target is determined by the location of two previous targets, with the locations of every three targets comprising a three-element fragment sequence^34^. There were 12 three-element fragment sequences in the SOC rule of the present study.

In the 850 ms SOA condition, stimulus presentation time was 600 ms, with an interval of 250 ms. In the 1350 ms SOA condition, stimulus presentation time was 600 ms and the interval was 750 ms. During the interval, four hollow circles were displayed on the screen. Participants were instructed to focus on these hollow circles and press the corresponding key to the solid circle (i.e., target) when it appeared by covering one of the hollow circles. Participants’ reaction time and accuracy were recorded.

### Experimental designs

A 2 (SOA: 850 ms vs. 1350 ms) × 3 (Training phase: early, middle, and late phase) mixed ANOVA design was adopted. SOA was a between-group variable and Training phase was a within-group variable. The experiment included a training stage and a test stage. Several dependent variables were used, including reaction time in the training stage, correct rate of the inclusion task and error rate of the exclusion task in the test stage (see Experimental procedure), and brain activation levels corresponding to each training phase.

In order to be fit with the fMRI design, two fixed SOAs (850 ms and 1350 ms) were used in the present study to ensure equal learning time for participants in both SOA groups. A pilot behavioral study was conducted before the formal test. The results showed that participants’ awareness of the implicit sequence rule under the two SOA conditions was an interim state lying between a fully unconscious state and a fully conscious state. However, participants’ awareness was more conscious in the 1350 ms SOA condition than that in the 850 ms SOA condition.

Traditionally, a random sequence block is inserted in implicit sequence training stage for measuring learning effect^35–37^. However, the literature has shown that the insertion of a random sequence block in implicit sequences often causes an artificially aggrandized learning effect due to its unexpected unpredictability^38–41^. Therefore, random sequence block was not used in the present study for gauging learning effect, which was measured by the inclusion and exclusion tasks (see Experimental procedure) instead.

### Experiment Procedure

The experiment included a training stage and a test stage. A practice session with 24 trials (in which locations of targets were randomized) was provided for participants to get familiarized with the test. Afterwards, participants started the formal experiment.

**Figure 1:**
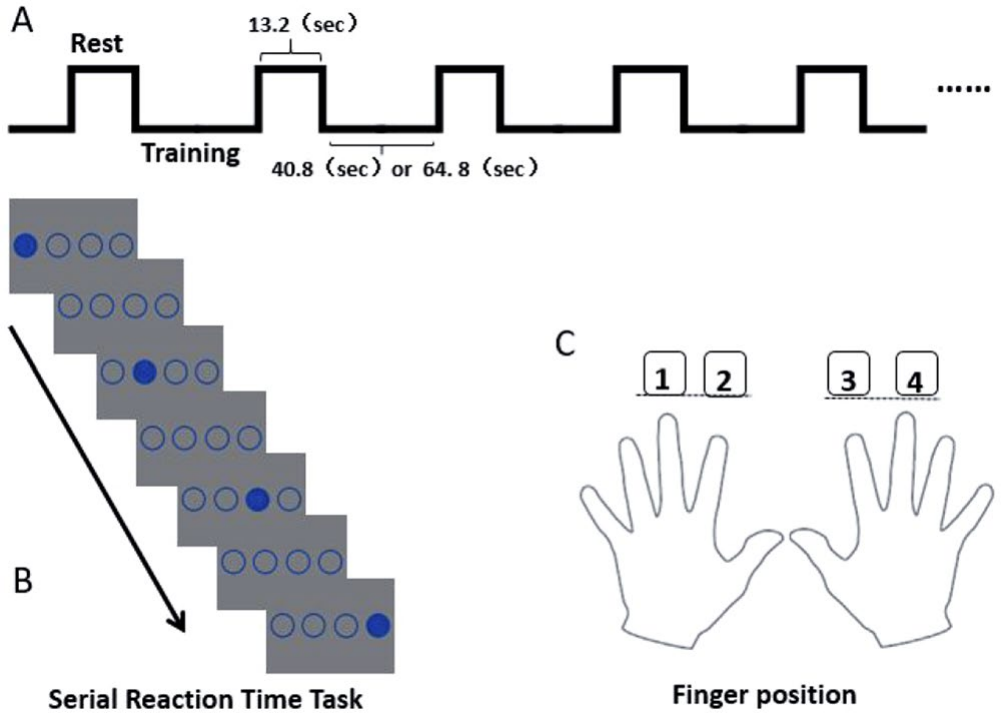
Experimental procedure. Note: (**A**) = block design in training stage; (**B**) = sample procedure for a single fMRI scan; (**C**) = corresponding key-press in the training stage.

A block design was used for fMRI scanning (see Fig. 1). The training stage was divided into 3 phases (5 blocks for each, with 48 trials in each block): phase 1 (block 1–5), phase 2 (block 6–10), and phase 3 (block 11–15). One scan was conducted for each training phase (240 trials). The interval between every two scans was 45 s. A resting block (13.2 s) was set before each training block, which was used as the baseline. The total scanning time was 15 minutes for the 850 ms SOA group, and 21 minutes for the 1350 ms SOA group.

After fMRI scanning on the training stage, participants were introduced to the test stage in a behavioral experimental room. An inclusion and an exclusion task were used. For the inclusion task, after viewing every two trials, participants were asked to predict the location of the target in the third trial, based on their knowledge obtained in the training stage. For the exclusion task, after viewing every two trials, participants were asked to point out the impossible locations of the target in the third trial. Both consciousness (explicit knowledge) and unconsciousness (familiarity) contribute to the accuracy rate of the inclusion task. In contrast, only unconsciousness contributes to the error rate of the exclusion task. Therefore, scores of consciousness were calculated by subtracting the error rate of the exclusion task from the correct rate of the inclusion task (inclusion_correct_ − exclusion_error_). There were 72 three-element fragment sequences in each task, and the random-choice rate for each task was 33.3% (note that the participants were not allowed to press the same key in succession).

### fMRI image acquisition

Images were acquired with a Siemens Trio 3.0 T scanner in the Laboratory of Cognition and Personality in Southwest University (China). Functional data were acquired in an interlaced way along the AC–PC line with a T2-weighted EPI sequence of 24 axial slices (TR = 1500 ms, TE = 30 ms, flip angle = 90°, acquisition matrix = 64 × 64, thickness = 5 mm, inter-slice gap = 1 mm). Within each session, a total of 644 EPI images were acquired. At the end of the experiment, a T1-weighted spin echo data set (TR = 2000 ms, TE = 2.52 ms, flip angle = 90°, acquisition matrix = 256 × 256) was acquired.

### Imaging data analysis

All images were analyzed using SPM8 (http://www.fil.ion.ucl.ac.uk/spm/software/spm8/). The first five volumes of each run were excluded from the analysis to allow for signal stability following onset transients. Functional images were corrected for differences in slice-timing, realigned, and co-registered with the structural images. These images were then normalized to the MNI template brain (voxel size: 3 × 3× 3 mm^3^), and smoothed with a Gaussian kernel (6 × 6 × 6 mm^3^ full-width, half-maximal).

The pre-processed imaging data were further analyzed with the use of general linear model (GLM). On the individual level, separate analyses were done for each of the three training phases, in which resting block and training block were convolved with canonical hemodynamic response function (HRF), yielding a main effect (R1, R2, and R3) for resting block (“1, 0” comparisons) and a main effect (S1, S2, and S3) for training block (“0, 1” comparisons) in each training phase.

On the group level, the analyses were conducted in two steps. In the first step, beta values of significantly activated brain regions (in comparison to resting baseline) for each training phase were extracted via the Marsbar software. A correlation analysis was conducted between these beta values and awareness scores (i.e., consciousness and unconsciousness levels as measured by the inclusion and exclusion tasks). In the second step, Bonferroni-corrected paired *t* tests were conducted to search for brain regions that significantly differed in activation levels between paired training phases (i.e., phase 2 vs. phase 1, phase 3 vs. phase 1, and phase 3 vs. phase 2). Activation difference scores (beta values) of these brain regions were calculated for each pair of training phases (e.g., beta_phase2_-beta_phase1_ for a given brain region), and then correlated to corresponding awareness scores. Statistical significance level was set as *p* < 0.001 (unc). Activated brain regions had to contain at least 20 voxels to be considered as meaningful.

## Results

### Behavioral Data

Bonferroni-corrected paired *t* tests for averaged reaction time differences between the two experimental groups showed that the 1350 ms SOA group took significantly longer to respond than the 850 ms SOA group did: *t* (43) = 13.67, *p* < 0.001. In the present study, reaction time differences between block 1 and block 15 were not applicable as a measure of learning effect because it was impossible to eliminate the effects of practice and fatigue. Neither could other traditional measures of learning effect (e.g., reaction time differences between standard triplet and deviant triplet, or between regular sequence block and random sequence block) be applicable due to the present experimental design. Therefore, learning effect was inferred by measures of awareness in the test stage.

**Figure 2:**
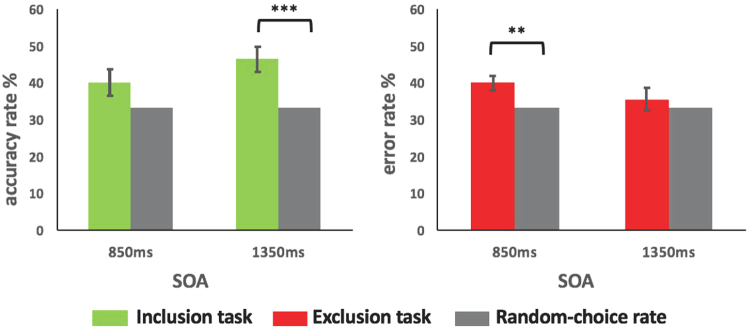
Comparisons of scores of inclusion and exclusion tasks to ratios of random choices for the 850 ms and 1350 ms SOA conditions. Note: **significant difference with *p* < 0.01; ***significant difference with *p* < 0.001.

According to the logic of the PDP paradigm, if correct rates are higher than random choice rates in the inclusion task, participants’ response is inferred as a result of collaboration between unconsciousness and consciousness. If error rates are higher than random choice rates in the exclusion task, participants’ response is inferred as a result of unconsciousness. The effect of consciousness in implicit processing is conventionally calculated by subtracting the error rate of the exclusion task from the correct rate of the inclusion task (i.e., inclusion_correct_ − exclusion_error_)^42^. Following this procedure, Bonferroni-corrected paired *t* tests were conducted to compare participants’ correct rates to random choice rates in the inclusion task, and participants’ error rates to random choice rates in the exclusion task (see Fig. 2). It was found that, for participants in the 850 ms SOA group, their correct rates were not significantly higher than random choice rates [*t*(44) = 1.87, *p* > 0.05] in the inclusion task, but their error rates were significantly higher than random choice rates [*t*(44) = 3.25, *p* < 0.01] in the exclusion task. These results suggest that only unconscious processing occurred in the 850 ms SOA group. For participants in the 1350 ms SOA group, their correct rates were significantly higher than random choice rates [*t*(42) = 3.86, *p* < 0.001] in the inclusion task, but their error rates were not significantly higher than random choice rates [*t*(42) = 0.76, *p* > 0.05] in the exclusion task. These results suggest that only conscious processing occurred in the 1350 ms SOA group. Different awareness states found for the two SOA groups suggested that awareness levels in implicit learning was sensitive to variation in SOA settings.

The two SOA groups were further compared by Bonferroni-corrected independent *t* tests on their scores of the inclusion task (correct rates), scores of the exclusion task (error rates), and scores of consciousness (inclusion_correct_ − exclusion_error_). No significant results were found [*t*_inclusion_ (43) = −1.25, *p* > 0.05; *t*_exclusion_(43) = 1.19, *p* > 0.05; *t*_inclusion-exclusion_(43) = −1.43, *p* > 0.05]. However, as can be seen in Fig. 2, the two groups showed a differed pattern on scores of consciousness, with the 1350 ms SOA group possessed a higher level of consciousness (though not statistically significant).

### fMRI results

#### Correlation analyses between brain activation levels and awareness scores

To explore the relationship between brain activation and awareness states, beta values of significantly activated brain regions (compared to the baseline of resting baseline) in each training phase were correlated to awareness scores (see Table 2), with the statistical significance Bonferroni-corrected. In the results, no significant correlation was found for the 850 ms SOA group. For the 1350 ms SOA group in training phase 2, beta values of left inferior parietal lobule positively correlated to scores of the inclusion task (*r* = 0.44, *p* < 0.05) and scores of consciousness (inclusion_correct_ − exclusion_error_) (*r* = 0.45, *p* < 0.05), but not to the scores of the exclusion task, suggesting that activation in this brain region was related to increased conscious processing. Beta values of left middle frontal gyrus positively correlated to the scores of the inclusion task (*r* = 0.43, *p* < 0.05), but not to the scores of the exclusion task or to scores of consciousness, suggesting that activation in this brain region was related to increased coordinated conscious and unconscious processing. Beta values of right inferior parietal lobule negatively correlated to scores of exclusion (*r* = −0.45, *p* < 0.05), but not to the other two indices, suggesting that activation in this brain region was related to reduced unconscious processing. For the 1350 ms SOA group in training phase 3, beta values of left lingual gyrus (*r* = −0.48, *p* < 0.05) and left inferior frontal gyrus (*r* = −0.45, *p* < 0.05) negatively correlated to scores of exclusion, suggesting that activation of these two brain regions was related to reduced unconscious processing. Beta activation of left inferior parietal lobule positively correlated to scores of consciousness (*r* = 0.63, *p* < 0.01) and negatively to scores of exclusion (*r* = −0.77, *p* < 0.001), suggesting that activation of this brain region was related to reduced unconscious processing and increased conscious processing at the same time.

**Table 2:** Correlation of beta values of brain regions activated in each training phase and awareness scores.

	Brain region	(SOA = 850 ms)			Brain region	(SOA = 1350 ms)		
		In	Ex	In-Ex		In	Ex	In-Ex
Phase1	r Inferior Frontal Gyrus	0.10	0.06	0.05	l Cuneus	0.17	−0.02	0.11
	l Lingual Gyrus	0.16	−0.12	0.17	r Lingual Gyrus	0.09	−0.26	0.20
	r Middle Frontal Gyrus	0.16	0.04	0.11	l Inferior Frontal Gyrus	0.34	−0.18	0.30
	l Precentral Gyrus	−0.16	0.21	−0.21	r Precentral Gyrus	0.09	−0.14	0.13
Phase2	r Inferior Frontal Gyrus	0.26	−0.25	0.31	l Lingual Gyrus	0.06	−0.13	0.11
	r Lingual Gyrus	0.24	0.09	0.15	l Inferior Parietal Lobule	**0**.44*	−0.35	**0**.45*
	l Middle Frontal Gyrus	0.16	0.18	0.05	l Middle Frontal Gyrus	**0**.43*	−0.24	0.39
					r Sub−Gyral	0.24	−**0**.45*	0.39
Phase 3	r Lingual Gyrus	0.22	0.11	0.12	l Lingual Gyrus	0.11	−**0**.48*	0.32
	l Middle Frontal Gyrus	0.01	0.19	−0.08	l Inferior Frontal Gyrus	0.19	−**0**.45*	0.35
	r Inferior Frontal Gyrus	0.28	0.00	0.22	l Inferior Parietal Lobule	0.36	−**0**.77**	**0**.63**

Note: l = left; r = right; In = correct rates of the inclusion task; Ex = error rates of the exclusion task; In-Ex = scores of consciousness; *p < 0.05; **p < 0.001.

Overall, the above findings showed that, for the 1350 ms SOA group, the function of left inferior parietal lobule was important both in the middle stage of training (i.e., perhaps mediating increased conscious processing) and in the late stage of training (i.e., possibly mediating reduced unconscious processing and increased conscious processing at the same time).

#### Correlation analyses between brain activation difference scores of paired training phases and awareness scores

In this part of the analysis, significantly activated brain regions in each training phase were subtracted respectively for the two SOA conditions. To closely examine whether each training phase was mediated by different brain networks, direct comparisons of activation differences between paired training phases would be useful. Therefore, Bonferroni-corrected paired *t* tests were conducted to search for brain regions showing significant activation differences between paired training phases (P2-P1, P3-P2, P3-P1). In the results, for the 850 ms SOA group, left medial frontal gyrus (activation increased), right middle frontal gyrus (activation increased), left superior frontal gyrus (activation increased), and right sub-gyral (activation decreased) were found to show significant activation differences for P2-P1 comparison. Moreover, left superior frontal gyrus (activation increased) and right cerebellum anterior lobe (activation decreased) showed significant activation differences for P3-P1 comparison. No significant regional activation difference was found for P3-P2 comparison. For the 1350 ms SOA group, left superior frontal gyrus (activation increased) and left putamen (activation decreased) were found to show significant activation differences for P2-P1 comparison. Left middle temporal gyrus (activation increased), left superior frontal gyrus (activation increased), left putamen (activation decreased), right putamen (activation decreased), and left superior temporal gyrus (activation decreased) showed significant activation differences for P3-P1 comparison. No significant regional activation difference was found for P3-P2 comparison. In these result, it could be seem that, for both the 850 ms and 1350 ms SOA group, brain regional activation showed significant differences between phase 2 and phase 1, but not between phase 3 and phase 2, suggesting that phase 2 was the critical period in which qualitative changes of implicit learning process occurred.

**Figure 3:**
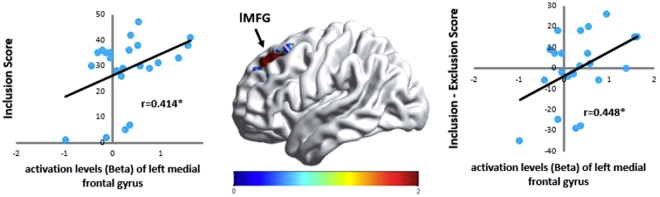
Patterns of correlation between difference beta values (P2-P1) and awareness scores over left medial frontal gyrus for the 850ms SOA group.

Moreover, difference beta values were calculated for brain regions showed significant activation differences between paired training phases, and then correlated to awareness scores. In the results, difference beta values of left medial frontal gyrus (P2-P1) positively correlated to scores of the inclusion task (*r* = 0.41, *p* < 0.05) and scores of the exclusion task (*r* = 0.45, *p* < 0.01) for the 850 ms SOA group (see Fig. 3). No such effect was found in the 1350 ms SOA group. These results suggested that left medial frontal gyrus only participated in consciousness development of implicit learning when consciousness levels were low.

## Discussion

Similar to previous reports that different states of awareness could be observed in different RSI settings during implicit learning^14,15^, the two SOA settings in the present study were found to produce two different states of awareness. Implicit sequence learning in the 850 ms SOA group was predominantly unconscious (i.e., as measured by error rates of the exclusion task). The reason for the absence of conscious processing in this group is probably due to the interference of wrong or irrelevant explicit knowledge on participants’ learning process. In contrast, implicit sequence learning in the 1350 ms SOA group was predominantly conscious (i.e., as measured by inclusion_correct_ − exclusion_error_). Comparison of participants’ awareness scores to random choice rates further showed that different SOA settings correlated to qualitatively different awareness states, with unconsciousness emerged in the 850 ms SOA group and consciousness in the 1350ms SOA group. Both the unconscious and conscious components are inter-states between full unconsciousness and full consciousness. These results supported the view of a dynamic gradual change of awareness in implicit learning. Notably, Zhang and colleagues proposed a dual-system evolving theory, in which representation quality and awareness state are thought as two different but related systems^43^. Awareness state changes with the improvement of representation quality, and the accumulation of such change can eventually cause it to evolve from unconsciousness to consciousness. However, in this evolving process, most of the time, an awareness state lies between full unconsciousness and full consciousness, with its transition mediated by different neural substrates. Based on this theory and the results of the present study, it may be expected that in conditions with SOAs vacillating around 850 ms, unconsciousness would be the predominant awareness state in implicit learning, with its contribution ratios varying subtly in accordance to SOA length changes. In contrast, conditions with SOAs vacillating around 1350 ms may produce predominantly an awareness state of consciousness.

In our fMRI results, inferior parietal lobule and frontal lobe were found to show continuously increased activation during the whole process of implicit sequence learning. This phenomenon may be explained by the theory of representation quality, that is, representation quality of implicit knowledge improves with the progress of learning. Inferior parietal lobule and frontal lobe have been found to play important roles in mediating the implicit knowledge acquisition process^30,44^. Therefore, continuously increased activation in these two brain regions over the whole course of implicit sequence learning suggests continually improving quality of implicit knowledge representation.

Putamen has been argued as a critical brain region mediating implicit motor sequence learning^27–29^. Moreover, decreased activation of putamen has been found to significantly correlate to the progress of implicit sequence learning^45^. In the present study, activation of putamen decreased when consciousness contribution to implicit learning increased, but only under long SOA condition. It has been found that putamen plays a critical role in location prediction for stimulus-response connection learning^46^. Accordingly, our finding may indicate that, in long SOA condition, location prediction function of putamen is important for early phase of implicit learning, but not for late phase, in which consciousness overwhelms unconsciousness and knowledge of implicit rule becomes more explicit than implicit.

To explore how specific brain activation pattern in each training phase and activation differences between paired training phases relate to awareness states, we conducted a series of correlation analyses. These analyses showed that, for the 850 ms SOA condition, brain regional activation were not correlated to the three awareness indexes (i.e., error rates of the exclusion task, correct rates of the inclusion task, and scores of inclusion_correct_ − exclusion_error_). In the 1350 ms SOA condition, however, activation of inferior parietal lobule correlated positively to consciousness levels (i.e., inclusion_correct_ − exclusion_error_) during the middle training phase, positively to consciousness levels as well as negatively to unconsciousness levels (i.e., error rates of the exclusion task) during the late training phase. These patterns suggest that inferior parietal lobule mediates the transition from unconscious processing to conscious processing in implicit learning. Further, for both the 850 ms SOA and the 1350 ms SOA conditions, brain activation patterns differed distinctly between early training phase (i.e., P2-P1), but not between middle and late training phases (i.e., P3-P2), suggesting that there was a processing transition (i.e., perhaps a transition from a predominantly unconscious processing to a predominantly conscious processing) of implicit learning between early and middle training phases. Moreover, in the 850 ms SOA condition, activation levels of inferior frontal gyrus during early training phase positively correlated to consciousness levels. At the same time, behavioral data showed that unconscious processing dominated the implicit learning in the short SOA condition. Altogether, these results suggest that inferior frontal gyrus mainly mediate conscious processing, and even a short SOA could elicit partial conscious processing.

Notably, one of the shortcomings of the present study is that participants’ awareness states were not measured concurrently with fMRI data acquisition (i.e., no random sequences were included in the design). Therefore, the correlations between brain activation and awareness states could not be observed on-line, which were inferred based on a post-hoc measure of awareness states as well as reports from previous literature^30,31,47^ instead. Future studies may establish a direct link between brain regional activation and awareness state by using on-line measurements of awareness during fMRI scanning. Another limit of the present study is that only two SOA conditions were considered. Future studies adopting successively changing SOAs may help us gain a clearer picture of the gradual changing process of awareness states in implicit learning.

In summary, the 850 ms and 1350 ms SOA settings elicited two different awareness states (existing between full unconsciousness and full consciousness). The 850 ms SOA elicited a predominantly unconscious processing and the 1350 ms SOA a predominantly conscious processing, which suggests that awareness states between full unconsciousness and full consciousness can be qualitatively different. Frontal lobe and inferior parietal lobule were found to play important roles in the implicit sequence learning process. For the 850 ms SOA condition, in which awareness level was low (i.e., predominantly an unconscious processing), strengthened activation of the frontal lobe mediated the subtle increase of a partially conscious processing. For the 1350 ms SOA condition, in which awareness level was relatively high (i.e., predominantly a conscious processing), strengthened activation of inferior parietal lobule supported the increase of conscious processing (in both early and late stages of implicit learning) and the decrease of unconscious processing (in the late stage of implicit learning) at the same time.
